# Crystal Structure of a Ube2S-Ubiquitin Conjugate

**DOI:** 10.1371/journal.pone.0147550

**Published:** 2016-02-01

**Authors:** Sonja Lorenz, Moitrayee Bhattacharyya, Christian Feiler, Michael Rape, John Kuriyan

**Affiliations:** 1 California Institute for Quantitative Biosciences, University of California, Berkeley, California, United States of America; 2 Department of Molecular and Cell Biology, University of California, Berkeley, California, United States of America; 3 Rudolf Virchow Center for Experimental Biomedicine, University of Würzburg, Würzburg, Germany; 4 Howard Hughes Medical Institute, University of California, Berkeley, California, United States of America; 5 Department of Chemistry, University of California, Berkeley, California, United States of America; 6 Physical Biosciences Division, Lawrence Berkeley National Laboratory, Berkeley, California, United States of America; NCI-Frederick, UNITED STATES

## Abstract

Protein ubiquitination occurs through the sequential formation and reorganization of specific protein-protein interfaces. Ubiquitin-conjugating (E2) enzymes, such as Ube2S, catalyze the formation of an isopeptide linkage between the C-terminus of a “donor” ubiquitin and a primary amino group of an “acceptor” ubiquitin molecule. This reaction involves an intermediate, in which the C-terminus of the donor ubiquitin is thioester-bound to the active site cysteine of the E2 and a functionally important interface is formed between the two proteins. A docked model of a Ube2S-donor ubiquitin complex was generated previously, based on chemical shift mapping by NMR, and predicted contacts were validated in functional studies. We now present the crystal structure of a covalent Ube2S-ubiquitin complex. The structure contains an interface between Ube2S and ubiquitin in trans that resembles the earlier model in general terms, but differs in detail. The crystallographic interface is more hydrophobic than the earlier model and is stable in molecular dynamics (MD) simulations. Remarkably, the docked Ube2S-donor complex converges readily to the configuration seen in the crystal structure in 3 out of 8 MD trajectories. Since the crystallographic interface is fully consistent with mutational effects, this indicates that the structure provides an energetically favorable representation of the functionally critical Ube2S-donor interface.

## Introduction

It is now apparent that many important biological processes are governed by protein-protein interactions that are extremely weak and transient, and therefore difficult to characterize. The ubiquitin cascade, a series of protein transfer reactions that regulates diverse physiological processes, provides a striking example of this concept [1]. In the course of the ubiquitination cascade, ubiquitin is passed from a ubiquitin activating enzyme (E1) to a ubiquitin conjugating enzyme (E2), and finally to a target protein, as catalyzed by a ubiquitin ligase (E3) [2]. Due to the transient nature of the underlying protein-protein interactions, it is challenging to obtain direct structural information for intermediate steps of the catalytic cascade, such as the recognition of target proteins by E3 enzymes or the interactions between E2 enzymes and ubiquitin. However, with the application of sensitive experimental techniques and computational methods considerable information is now emerging on these intermediates.

In this paper we analyze the interaction between a particular E2 enzyme, Ube2S, and a ubiquitin molecule that is covalently linked to the E2 active site. Together with its cognate E3 enzyme, the human anaphase-promoting complex/cyclosome (APC/C), Ube2S forms Lys 11-linked ubiquitin chains on mitotic regulators to trigger their proteasomal degradation [3–5]. Ube2S has an inherent specificity for the formation of Lys 11-linkages, even in the absence of the APC/C [3,6,7]. Nevertheless, the interactions between Ube2S and its ubiquitin substrates are weak and at the limit of direct experimental detection [6].

**Fig 1:**
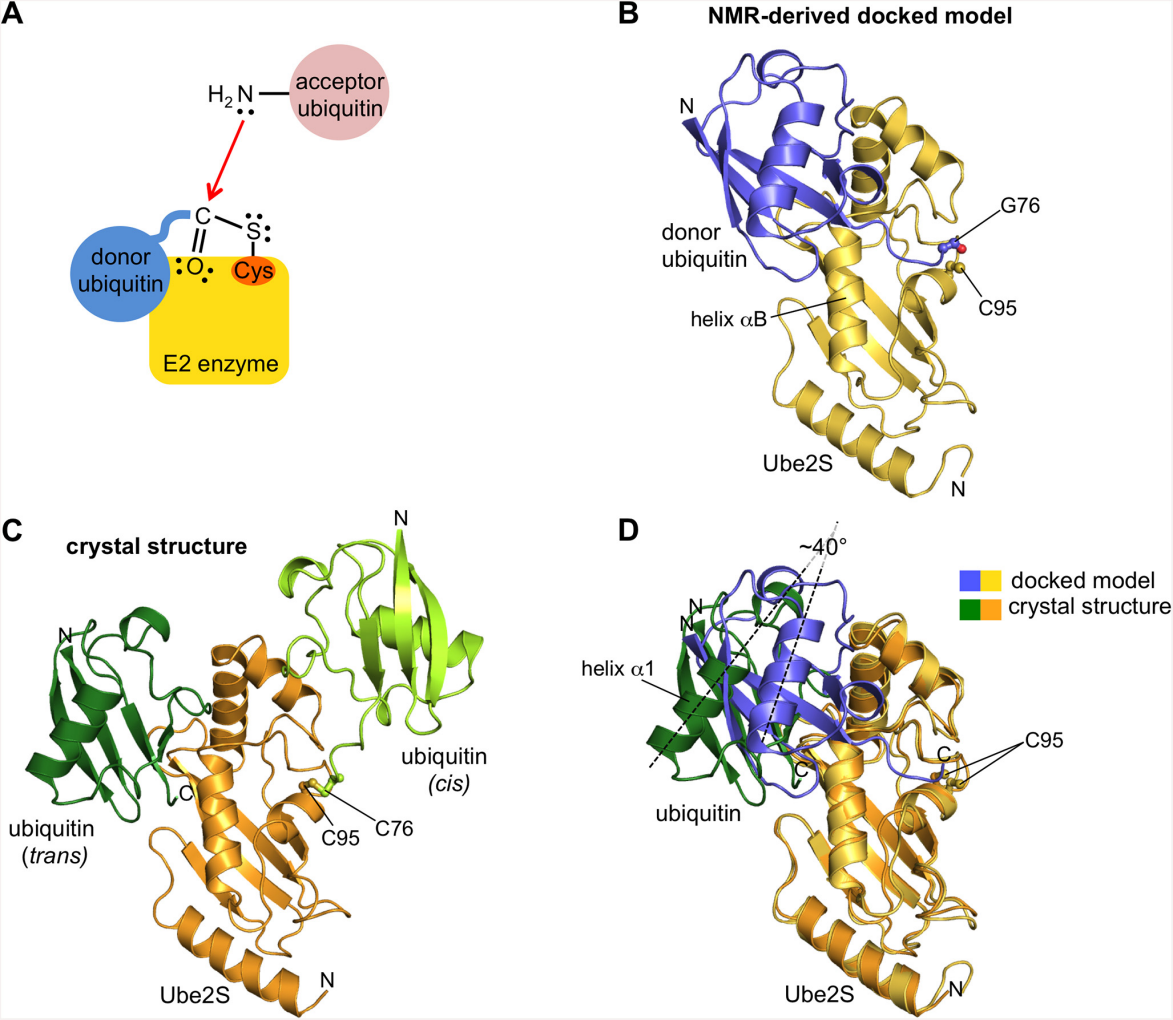
Schematic, NMR-derived docked model, and crystal structure of the Ube2S-ubiquitin conjugate. (A) Schematic of the critical step in ubiquitin linkage formation by E2 enzymes. The C-terminal carbonyl group of a donor ubiquitin that is thioesterified with the E2 active site cysteine undergoes a nucleophilic attack by a primary amino group of an acceptor ubiquitin. As a result, an isopeptide bond between donor and acceptor ubiquitin is formed. (B) NMR-derived docked model of the Ube2S-donor ubiquitin complex [6]. The catalytic UBC domain of Ube2S (yellow) and ubiquitin (blue) are shown in cartoon representation. The C-terminal carbonyl group of ubiquitin and the catalytic cysteine side chain of Ube2S are displayed in ball-and-stick mode. (C) Crystal structure of Ube2S C118M (orange) disulfide-linked to ubiquitin G76C (light green). The cysteine side chains forming the disulfide linkage are highlighted in ball-and-stick mode. Note that contacts between the two proteins (in cis) are limited to the vicinity of the disulfide linkage. In the crystal, however, Ube2S forms an interface with a second ubiquitin molecule (dark green) in trans. (D) The crystallographic complex formed in trans between Ube2S (orange) and a neighboring ubiquitin molecule (green) is superposed with the NMR-derived docked model of Ube2S (yellow) and donor ubiquitin (blue) [6]. The catalytic cysteine side chains are shown in ball-and-stick rendition. The angle by which the axes of helix α1 of ubiquitin are pivoted with respect to each other in the two configurations is indicated.

The critical step in the formation of ubiquitin linkages is a nucleophilic substitution reaction, in which the ε-amino group of a lysine residue (or the N-terminal amino group) of an “acceptor” ubiquitin molecule attacks the C-terminal carboxyl group of a “donor” ubiquitin that is linked through a thioester bond to the active site cysteine of the E2 enzyme (Fig 1A) [8]. The result is an isopeptide (or peptide) linkage between the donor and the acceptor ubiquitin and, through iterations of this reaction cycle, the formation of a ubiquitin chain. The specificity of linkage formation is determined by which primary amino group of the acceptor ubiquitin acts as a nucleophile at the E2 active site. In contrast, the positioning of the donor ubiquitin on the E2 impacts the efficiency and processivity of chain formation [6,9–16].

The interactions between the donor ubiquitin and various E2 enzymes have been characterized extensively [6,9–31]. A comparison of the available structures for E2-donor ubiquitin conjugates [10,12–14,17,19,20,22,25–30] along with NMR, small angle X-ray scattering, and docking analyses [6,9,11,16,18,21,23,24] shows that the donor ubiquitin, while being covalently bound at the active site of E2 enzymes, is typically flexible and can adopt a wide range of different orientations, depending on the particular E2 enzyme, the experimental conditions, and the presence or absence of various binding partners. A subset of “closed” configurations, in which the hydrophobic patch of the donor ubiquitin contacts helix αB of the E2 enzyme, have emerged as catalytically poised states that appear to be conserved topologically across the E2 family [6,9–16,18,21,29–31]. However, the interfacial residues on the E2 enzymes are poorly conserved, and so the closed E2-donor interfaces vary in structural detail [12]. Closed configurations have also been observed for an E2 conjugate containing the ubiquitin-like modifier Nedd8 [32] and complexes of substrate-bound SUMO1 with its corresponding E2 and E3 enzymes [33,34].

Ube2S and ubiquitin adopt a closed interface, even when mixed in trans and in the absence of the APC/C [6]. However, with K_D_ values of 1.1 mM and 1.7 mM for the catalytic domain and full-length Ube2S, respectively, this non-covalent interaction is extremely weak [6]. Residues at the interface between Ube2S and donor ubiquitin were identified previously from chemical shift perturbation analysis by nuclear magnetic resonance (NMR) spectroscopy [6]. Based on these restraints a model of a closed Ube2S-donor ubiquitin complex was generated using computational docking (Fig 1B) and was validated by mutagenesis in combination with activity assays [6]. An inherent limitation of this approach is that direct experimental measurements of interatomic distances could not be obtained, and thus did not enter the docking calculation. Instead, restraints on the docking were provided only in the form of expected residue proximities. Thus it is not clear how precise or accurate the resulting model is in detail, although the fact that it explains a wide range of mutational data means that the essence of the functionally relevant interface is captured.

Here we report a crystal structure of a Ube2S-donor ubiquitin complex that contains a hydrophobic, closed interface between the two proteins in trans. Our analysis indicates that this interface provides an energetically favorable configuration compared to the earlier docked model and satisfies all functional restraints available from mutagenesis studies.

## Materials and Methods

### Protein preparation and crystallization

The previous [6] and present structural analyses of the Ube2S-donor interaction are restricted to the catalytic UBC domain of Ube2S (residues 1–156), thus excluding a flexible 66-residue C-terminal extension. To prepare a homogeneous disulfide-linked complex between the UBC domain and ubiquitin [24] we first generated the single-cysteine mutants Ube2S C118M (as well as the C118S and C118A) and ubiquitin G76C by ligation-during-amplification approaches [35].

All proteins were produced recombinantly in *Escherichia coli* from a modified His_6_/SMT3-encoding plasmid (Ube2S) [36] and a pet30a expression vector (ubiquitin), respectively, as was described previously [6]. The corresponding purification strategies have also been described [6]. In short, Ube2S variants were purified by immobilized metal affinity chromatography (HisTrap HP, GE) and gel filtration (Superdex 75pg, GE), and ubiquitin was isolated from the cell lysate by perchloric acid precipitation, cation exchange chromatography (HiTrap SP HP, GE), and gel filtration (Superdex 75pg, GE). The final purification steps were performed in 50 mM sodium phosphate, pH 7.5. To generate a disulfide linkage between the purified protein variants we first activated Cys 76 of ubiquitin by DNTB (5,5’-dithio-bis-nitrobenzoic acid), removed excess DNTB through buffer exchange, and then added a sub-stoichiometric amount of the Ube2S C118M variant. DTNB released upon the disulfide exchange reaction was removed through buffer exchange, and the disulfide-linked protein complex was isolated from excess ubiquitin by anion exchange chromatography using a UnoQ column (BioRad) with a gradient of 25 to 500 mM NaCl in 25 mM Tris, pH 7.5. The purified complex was then concentrated to 50 mg/ml and crystallized in sitting drops of 0.1 M Tris, pH 8.0, and 20% PEG 4000 at 20°C. The protein crystals were cryo-protected in 0.1 M Tris, pH 8.0, 20% PEG 4000, and 25% ethylene glycol before freezing.

### Crystallographic data collection and processing

X-ray diffraction data were collected at the Stanford Synchrotron Radiation Lightsource, beam line 11–1 and were processed with XDS [37]. Molecular replacement was performed with Phaser [38] as implemented in the CCP4 (collaborative computational project no. 4) suite [39] using PDB ID 1ZDN [40] as a search model. Refinement was performed using Phenix [41] and model building was done in Coot [42]. Atomic coordinates and structure factors have been deposited in the protein data bank under PDB ID: 5BNB.

### Activity assays

In vitro activity assays monitoring diubiquitin formation were performed as described previously [6]. The reactions contained 0.25 μM E1 enzyme, 5 μM E2 enzyme, 60 μM ubiquitin, 3 mM ATP, and 7.5 mM MgCl_2_.

### Molecular dynamics simulations

The molecular dynamics trajectories were generated using the Gromacs 4.6.2 package [43,44] and the ff99SB-ILDN force field [45]. All simulations were carried out in aqueous medium using the TIP3P water model and appropriate counter ions (Na^+^ and Cl^-^) were added to neutralize charges. After initial energy minimization, the systems were subjected to 100 ps of constant number, volume and temperature (NVT) equilibration, during which the system was heated to 300K. This was followed by a short equilibration at constant number, pressure and temperature (NPT, 100 ps). The equilibration steps were performed with harmonic positional restraints on the protein atoms. Finally, the production simulations were performed under NPT conditions, with the Berendsen and v-rescale thermostats in Amber12 and Gromacs 4.6.2 respectively, in the absence of positional restraints. Periodic boundary conditions were imposed, particle-mesh Ewald summations were used for long-range electrostatics, and the van der Waals cut-off was set to 1 nm. A time step of 2 fs was employed.

## Results and Discussion

### Crystal structure of a Ube2S-donor ubiquitin complex

To study the Ube2S-donor ubiquitin interaction by X-ray crystallography we stably linked the active site cysteine residue of Ube2S to an engineered cysteine at the C-terminus of ubiquitin by a disulfide bond [24]. This was necessary, since the interaction between Ube2S and ubiquitin in trans is likely too weak for co-crystallization [6], and the native thioester linkage as well as its oxy-ester analogue are susceptible to hydrolysis ([6] and data not shown). To obtain a homogeneous disulfide-linked conjugate, we utilized a single-cysteine containing variant of Ube2S, in which the second cysteine residue, Cys 118, had been substituted by methionine. We chose this particular variant, since it interacts with donor ubiquitin in the same way as the wildtype, as demonstrated by NMR chemical shift perturbation analysis (Figure A in S1 File). The C118M variant also displays the most wildtype-like, albeit reduced, activity in diubiquitin formation compared to other Cys 118 variants that we tested (Figure B in S1 File). It is currently unclear why the substitution of Cys 118 leads to a reduction in activity. Importantly for this study, however, the NMR analysis (Figure A in S1 File) and further structural data presented in this study demonstrate that the C118M substitution does not impact the binding mode of donor ubiquitin to Ube2S.

Hence, we crystallized the disulfide-linked Ube2S (C118M)-ubiquitin (G76C) complex and solved its structure by molecular replacement at 2.49 Å resolution (Table 1; data deposited under PDB ID: 5BNB).

**Table 1:** Structure determination and refinement.

**Data collection**	
Wavelength	0.979
Space group	P 1 21 1
Unit cell parameters	
a,b,c (Å)	94.9 45.9 110.6
α,β,γ (°)	90.0 93.1 90.0
Resolution (Å)	47.38–2.49 (2.59–2.49)
Total reflections	106496 (10254)
Unique reflections	32942 (3558)
R_pim_	3.6 (34.5)
Completeness (%)	97.4 (95.5)
I/σI	16.3 (2.2)
Redundancy	3.2 (2.9)
Wilson B factor	47.3
CC 1/2	0.99 (77.8)
**Refinement**	
Resolution (Å)	47.38–2.49 (2.56–2.49)
R_work_/R_free_ (%)	24.30 / 30.40
No. of atoms	6175
Protein	6105
Water	70
Average B-factors	72.7
Protein	72.8
Water	58.2
RMS deviations from ideality	
Bond lengths (Å)	0.002
Bond angles (°)	0.629
Ramachandran statistics	
Favoured (%)	96.01
Disallowed (%)	0
MolProbity overall score	1.17

Values in parentheses correspond to the highest shell.

There are four Ube2S-ubiquitin conjugates in the asymmetric unit. The Ube2S and ubiquitin molecules in these conjugates adopt similar orientations with respect to each other. Three E2 molecules and three ubiquitin molecules are relatively well-ordered. The fourth molecules of each kind have relatively poor electron density and could not be built completely. For two of the ubiquitin molecules, the C-terminal tails that connect to the E2 active site cysteine residues could be built and, considering the spatial arrangement of the remaining molecules in the crystal lattice, it is clear which Ube2S molecule is linked to which ubiquitin. Within each conjugate (in cis) only few contacts are formed between Ube2S and the donor ubiquitin (interface area ~ 180 Å^2^, Fig 1C), resulting in an open configuration. The contacts are located in the immediate vicinity of the disulfide bond and include Cys 95, Asn 97, Ser 127, and Leu 129 of Ube2S, and Arg 74, Gly 75, and Cys 76 of the ubiquitin tail, respectively.

**Fig 2:**
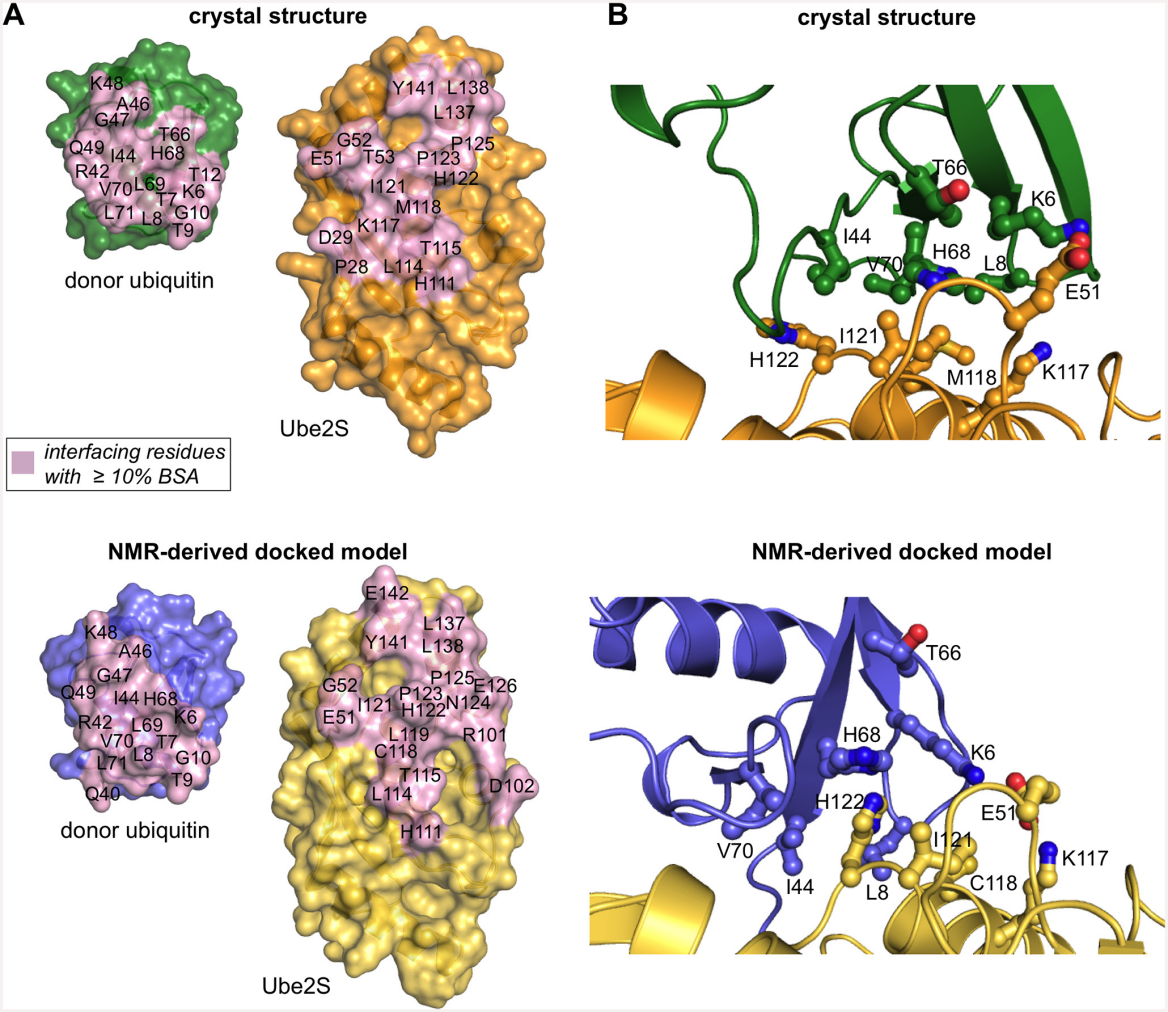
Comparison of donor recognition between the crystal structure and the NMR-derived docked model. (A) Open-book view of the interaction “footprint” on the surface of Ube2S and ubiquitin (residues 1–71) in the crystal structure (top) and in the docked model (bottom), respectively, as defined by residues that become ≥ 10% buried at the interface. The C-terminal tail of ubiquitin (residues 72–76) was omitted in this analysis and representation. (B) Details of the Ube2S-donor ubiquitin interfaces seen in the crystal structure (top) and the docked model (bottom), respectively. Key interfacial residues are displayed as balls-and-sticks. Note that Lys 117 of Ube2S and Thr 66 of ubiquitin do not make contacts in the docked model and are displayed for comparison only. Cys 118 is replaced by methionine in the crystal structure.

The hydrophobic surface patch on the globular core of ubiquitin, centered about Leu 8, Ile 44, and Val 70, instead forms an interface with a neighboring E2 molecule in trans (Figs 1C and 2). This interface resembles the one seen in the closed NMR-derived docked complex (Figs 1D and 2) [6]. This is illustrated by the footprints of the interacting surfaces of Ube2S and the globular core of ubiquitin, respectively, as defined by those residues whose surface area becomes buried by at least 10% upon complex formation (Fig 2A). Importantly, many key interfacial residues are preserved between the docked model and the crystal structure, such as Cys 118 (replaced by methionine in the crystallization construct), Ile 121, His 122, Pro 123, Leu 137, and Tyr 141 of Ube2S and Lys 6, Leu 8, Ile 44, Ala 46, Gly 47, Lys 48, His 68, and Val 70 of ubiquitin. These residues include the ones that were proven critical in activity assays and/or were identified by chemical shift perturbation analysis (S1 Table and S1 Fig) [6]. Notably, an ion pair between Glu 51 of Ube2S and Lys 6 of the donor ubiquitin that the docked model had predicted (Fig 2B) and that was functionally verified in charge-swap experiments [6] is also present in the crystal structure (Fig 2B) (S1 Table).

**Fig 3:**
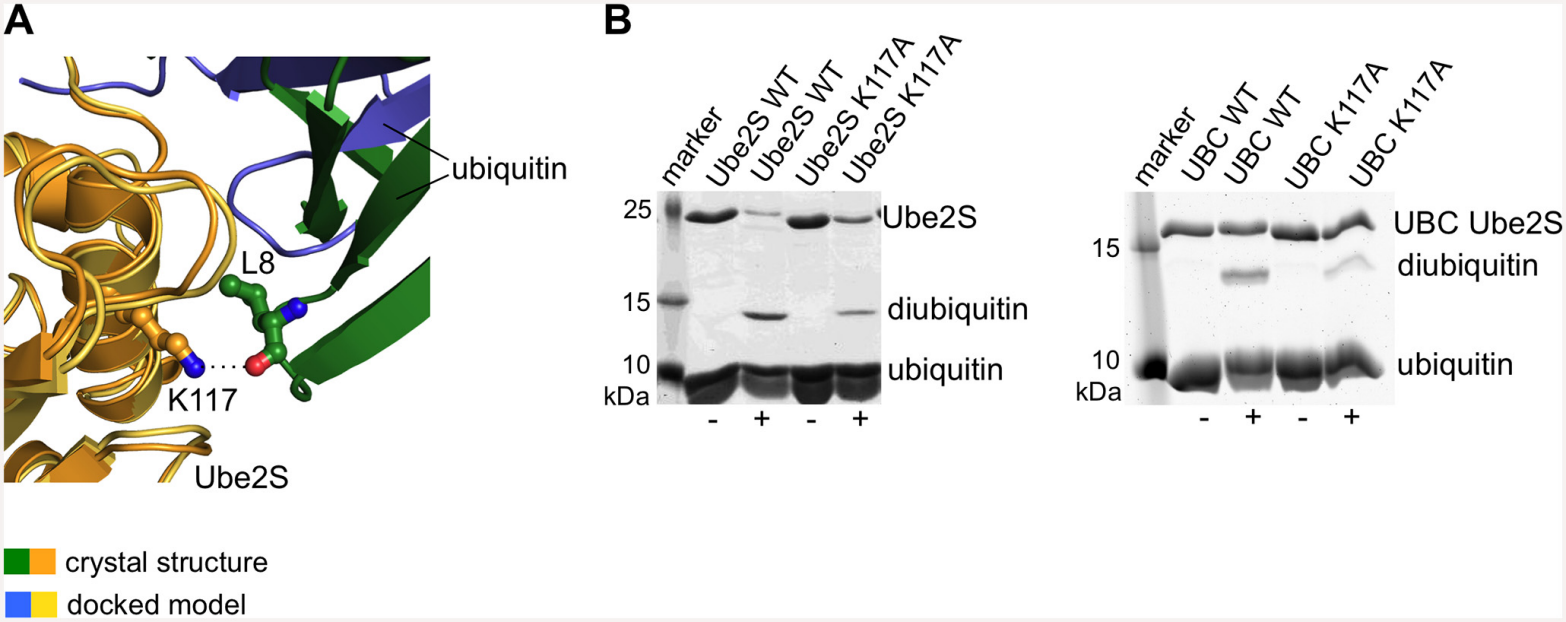
Effect of Lys 117 of Ube2S on activity. (A) Detail of the structural superposition of the docked model and the crystal structure provided in Fig 1D. The Ube2S molecules are shown in yellow and orange, respectively; the ubiquitin molecules in blue and green, respectively. In the crystal structure the sidechain amino group of Lys 117 of Ube2S forms a hydrogen bond with the backbone oxygen atom of Leu 8 of ubiquitin (as indicated by the dotted line), while Lys 117 is removed from the donor interface in the docked model (see Fig 2B). (B) In vitro activity assays monitoring diubiquitin formation by full-length Ube2S (left) and the UBC domain of Ube2S (right). We compared the corresponding wildtype proteins with the K117A variants in the absence (-) and presence (+) of ATP. The K117A substitution results in decreased diubiquitin formation.

While the contacting surfaces in both models are quite similar in their residue composition and size (~ 590 Å^2^ in the crystal structure versus ~ 510 Å^2^ in the docked model), the donor ubiquitin molecules are pivoted by ~ 40° with respect to each other (Fig 1D). As a result, the crystal structure has a more favorable packing of hydrophobic interfacial residues (Fig 2B), which is corroborated by interface scoring programs, such as PDBePISA (www.ebi.ac.uk/pdbe/pisa) that predict a significantly more negative solvation free energy gain upon complex formation for the crystal structure than for the docked model (-8.3 kcal/mol versus -0.9 kcal/mol). As will be discussed more in the following, the crystal structure may therefore provide a better representation of the Ube2S-donor interface than the docked model. In line with this idea, Thr 66 of ubiquitin that is part of the tightly-knit crystallographic interface with Ube2S is critical for donor ubiquitin function [6] (S1 Table). However, this residue does not make any contacts with Ube2S in the NMR-derived docked model (Fig 2B) (S1 Table). Also, Lys 117 of Ube2S forms a hydrogen bond with the backbone oxygen atom of Leu 8 of ubiquitin, while this hydrogen bond is not present in the docked model (Figs 2B and 3A). We thus tested the K117A variant of Ube2S in an activity assay that monitors diubiquitin formation and found that it exhibits reduced activity compared to the wildtype (Fig 3B). For a complete overview of mutational results mapped onto the crystal structure and the docked model, respectively, see S1 Fig.

We conclude that the hydrophobic interface observed in the crystal structure in trans can be viewed as a mimic of the functionally relevant interface between Ube2S and the globular core of donor ubiquitin. Consistent with this interpretation, the interface formed in the crystal in trans could theoretically also be formed within one Ube2S-donor conjugate in cis. In the crystal structure, the distance between the backbone nitrogen atom of residue Val 70 of ubiquitin and the sulphor atom of the active site, Cys 95, of Ube2S is ~ 20 Å. To provide a linkage in cis, this distance would need to be bridged by the seven C-terminal residues of ubiquitin. If the ubiquitin tail adopts a relatively extended conformation, this is possible. Rotamer changes and structural plasticity at the active site of Ube2S could make the distance to be bridged even smaller.

It should also be noted that the crystallographic Ube2S-donor interface that we present here is also compatible with the position of the Lys 11-specific acceptor ubiquitin that was proposed in a previous docked model of the ternary reaction complex [6].

We next analyzed if other contacts with neighboring molecules might influence the position of ubiquitin with respect to Ube2S in the context of the crystal lattice. As shown in Figure A in S2 File ubiquitin makes contacts with a total of five Ube2S molecules and another ubiquitin molecule, albeit to varying degrees. Three of those interfaces are even smaller than the one formed in cis (~ 30, ~ 90, ~ 160 Å^2^, respectively) and, therefore, likely energetically negligible (solvation free energy gain upon formation of the interface, Δ^i^G, of 0.1, -0.2, and 0.1 kcal/mol, respectively). The other two interfaces are the hydrophobic, closed interface that we have described above and an interface formed by residues in the N-terminal helix of Ube2S and Leu 8, Thr 9, Gln 31, Asp 32, Glu 34, Ile 36, Pro 37, Gln 40, and Leu 71 of ubiquitin (Figure B in S2 File). The latter interface is significantly smaller (~ 430 versus ~ 590 Å^2^) and less hydrophobic (Δ^i^G = -4.2 kcal/mol versus -8.3 kcal/mol) than the closed interface. To corroborate this analysis and to test if the hydrophobic trans interface that we have functionally validated is stable in the absence of other crystallographic lattice contacts we performed molecular dynamics (MD) simulations.

### Molecular dynamics simulations

We performed two series of MD simulations with the crystal structure and the docked model, respectively, using the Gromacs 4.6.2 package [43,44] and the ff99SB-ILDN force field [45]. For comparability the C-terminal tail of ubiquitin (residues 73–76) was excluded from both series of simulations. We carried out a total of five independent simulations of the crystal structure and eight of the docked model, over 100 ns each.

**Fig 4:**
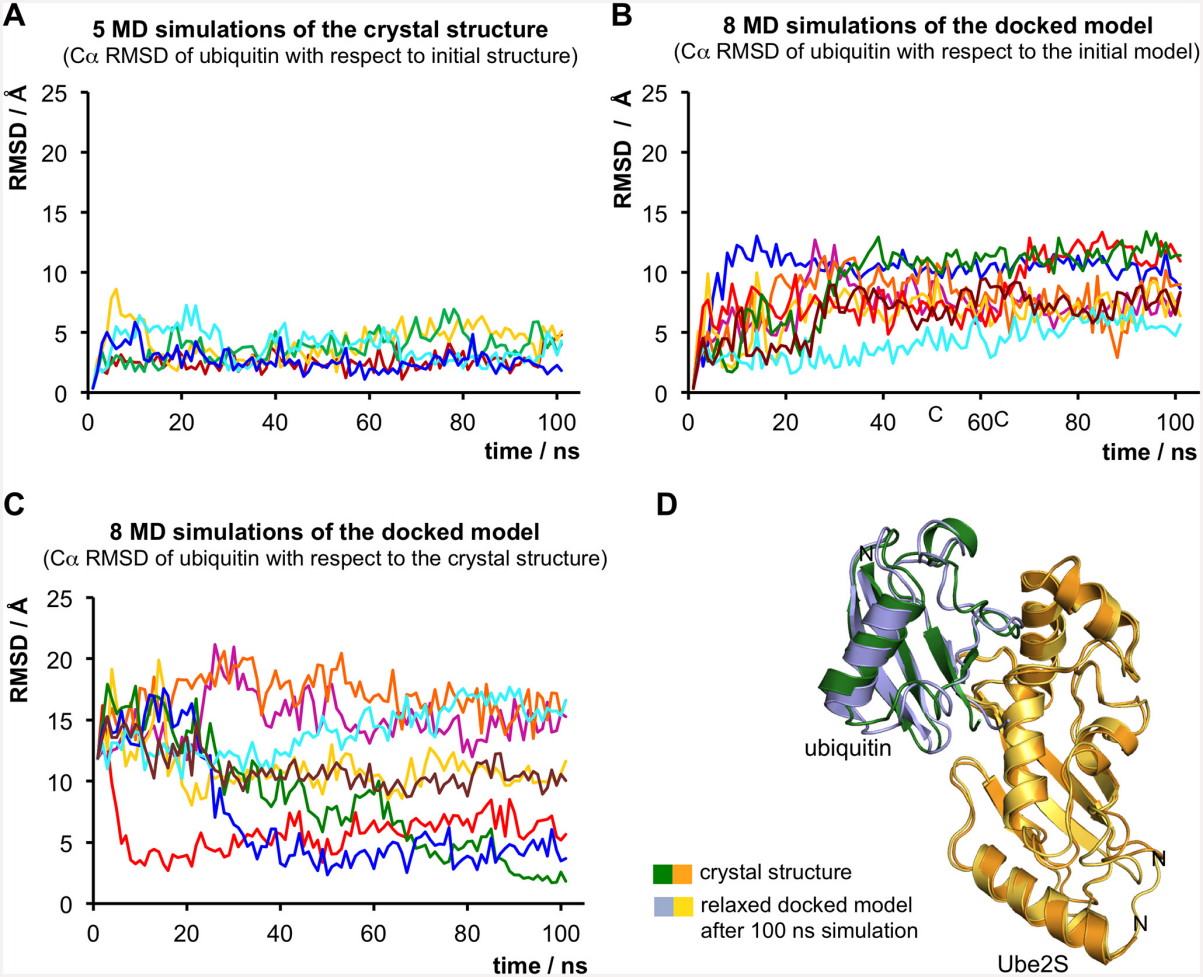
Molecular dynamics simulations. (A) Five independent trajectories (colored differently) of the crystal structure over 100 ns each. For each simulation the Ube2S molecules were aligned and the Cα RMSD values for ubiquitin (in Å) with respect to the crystal structure are plotted over the time. (B) Eight independent trajectories (colored differently) of the docked model over 100 ns each. For each trajectory the Cα RMSD values for ubiquitin (in Å) with respect to the starting model after aligning the Ube2S molecules are plotted. (C) For each trajectory of the docked model (see (B)), the Cα RMSD values for ubiquitin (in Å) with respect to the crystal structure after aligning the Ube2S molecules are plotted. In three trajectories (colored in red, dark blue, and green) the model converges to a configuration that is very similar to the crystal structure. (D) Superposition of the docked model after 100 ns of simulation time (see (B) and (C), green simulation run) with the crystal structure.

We found that the crystal structure is stable over the course of the simulations, with an overall average Cα RMSD for ubiquitin of ~ 3.4 Å from the starting structure, after aligning the Ube2S molecules (Fig 4A). In line with our analysis of the crystallographic lattice contacts (S2 File), this indicates that the interface between Ube2S and ubiquitin that we observed in the crystal structure in trans is not dominated by other lattice contacts with neighboring molecules, but represents a low-energy state, independent of the crystal environment.

In contrast, in each simulation of the docked model the trajectories deviate form the starting model, the overall average Cα RMSD for ubiquitin amounting to ~ 11.2 Å from the starting model, after aligning the Ube2S molecules (Fig 4B). These observations indicate that the docked Ube2S-ubiquitin interface is less stable than the crystallographic one.

Remarkably, we observe that the docked model quickly converges to the configuration seen in the crystal structure in three out of eight trajectories (Fig 4C). Fig 4D shows a representative example of the striking similarity of the crystal structure and the state that the simulated docked model relaxes to (Cα RMSD for ubiquitin ~ 1.8 Å after aligning the Ube2S molecules). That the MD trajectories starting from the docked model readily “find” the crystal structure suggests that the two configurations are close in conformational space and connected by a free-energy funnel and that the crystal structure presents a lower-energy configuration compared to the docked model.

It should be noted that the docked model was generated based on the crystal structure of wildtype Ube2S (PDB ID: 1ZDN) [40], i.e. it has the native cysteine residue at position 118. The convergence of the docked model to the crystallographic configuration (containing Ube2S C118M) during the MD simulations, therefore, provides additional support for notion that the low-energy crystallographic configuration is not a result of the C118M substitution.

Taken together, we conclude that the differences between the docked and the crystallographic configurations arise principally from the intrinsically lower accuracy of the docked model and that the crystal structure represents an energetically favorable Ube2S-donor ubiquitin interface.

## Conclusions

Here we present the crystal structure of a Ube2S-ubiquitin conjugate. The particular swung-out orientation of ubiquitin with respect to Ube2S seen in our crystal structure in cis has not been observed before and may be considered a snapshot of an open state with little contacts between the two proteins (though in the context of the crystal lattice, ubiquitin is held in place through its interaction with the neighboring Ube2S molecule in trans) (Fig 1C). In contrast, a hydrophobic interface formed between Ube2S and donor ubiquitin in trans mimics the catalytically important closed state and is in excellent agreement with all mutational data available for this system (S1 Table and S1 Fig). Strikingly, the previous docked model of the Ube2S-donor ubiquitin complex converges readily to the crystallographic configuration in MD simulations.

These observations demonstrate firstly that the docked model—despite having been generated without interatomic distance restraints in the form of NOE (Nuclear Overhauser Effect) measurements—is close in conformational space to the lower-energy crystallographic configuration. In line with this observation, both the docked model and the crystal structure have largely overlapping contact surfaces and are supported by extensive mutational data. Secondly, the differences between the docked model and the crystal structure are neither due to differences in environment (solution versus crystal) nor the amino acid substitution at position 118 of Ube2S, but they are likely due to the inherent imprecisions of the docking process. The Haddock docking protocol is followed by energy minimization procedures, but it does not include molecular dynamics [46]. Those, however, allow the docked model to sample conformational space more extensively and, therefore, enable it to “find” the energy minimum provided by the crystallographic configuration. Thirdly, the crystallographic interface presents a lower-energy configuration compared to the docked model and—despite being formed in the crystal in trans—it mimics the functionally relevant Ube2S-donor ubiquitin interface, as it is formed during catalysis when donor ubiquitin is covalently bound at the E2 active site.

S3 File shows how the Ube2S-ubiquitin configuration seen in our crystal structure compares to other structures of closed E2-donor complexes. Among those, very similar closed configurations are found for the donor conjugate of UbcH5A bound to the dimeric RING domain of RNF4 [10] (Figure A in S3 File), the donor conjugate of UbcH5B bound to the dimeric RING domain of BIRC7 [13], the donor conjugate of UbcH5B bound to CBL-B [14], the donor conjugate of UbcH5B bound to the RING domain of RNF38 [29], and the donor conjugate of Ubc13 bound to the RING domain of RNF4 and UbeV2 [30]. A slightly different ubiquitin orientation of ubiquitin is seen in a non-covalent and small-molecule bound complex of Cdc34 and ubiquitin (Figure B in S3 File). In this configuration as well as the aforementioned E3-containing conjugates, ubiquitin utilizes the same hydrophobic surface (including residues 6–9, 42, 44, 46–49, 68–76) to interact with the respective E2s as in our Ube2S-donor complex (Figure C in S3 File). However, these other structures differ from the closed Ube2S-donor orientation that we describe (crystal structure and Haddock model), in that the donor ubiquitin is tilted with respect to the E2 and shifted downwards along helix αB (S3 File). That the Ube2S-donor interaction is different from other systems is not unexpected, since Ube2S engages its cognate E3, the APC/C, in a non-canonical manner [3,6,47–49], and is activated by the RING-containing subunit (APC11) differently from other RING-E2 interactions [47,48]. Rather than stabilizing the closed donor configuration, APC11 has been shown to lower the K_M_-value for the acceptor ubiquitin [47,48]. Therefore, it is likely that the structural mechanisms that impact the closed Ube2S-donor ubiquitin configuration on the APC/C also differ from other E2/E3 systems. In the future it will be very interesting to explore how the closed Ube2S-donor orientation is stabilized in the context of the APC/C and how this donor orientation impacts the Ube2S catalytic center to enhance the accessibility and reactivity of the thioester bond. As our crystal structure has the relevant Ube2S-ubiquitin interface in trans, these structural details remain to be elucidated.

## Supporting Information

S1 Fig Mutational effects mapped onto the interfaces seen in the crystal structure and the docked model. The crystallographic (trans) and docked interfaces are displayed in open-book style, as in Fig 2A. Interfacial residues that were mutated and found to reduce activity in this study or previously [6] are encircled red. Residues whose substitution had no effect on activity are encircled white. Note that residues with ≥ 10% BSA (buried surface area; colored pink) do not necessarily make contacts that are energetically relevant for the interaction between Ube2S and donor ubiquitin. Lys 117 of Ube2S and Thr 66 of ubiquitin, both of which are important for activity, are interfacial residues in the crystal structure, but do not make contacts in the docked model (for details see text). Note that Arg 101 and Asp 102 of Ube2S, which are important for activity, are close to the active site and thus contact residues of the ubiquitin tail in the docked model. Since the crystallographic interface is formed in trans, i.e. the tail is oriented towards a neighboring E2 molecule, these Ube2S residues are not part of the interface in the crystal structure.
(TIF)

S1 FileEffects of Cys 118 substitutions in Ube2S on ubiquitin binding and activity.NMR data were recorded at 25°C on a Bruker 800 MHz DRX spectrometer, equipped with a ^1^H/^15^N/^13^C cryoprobe and were processed with NMRPipe [50]. The binding experiments were performed as described previously [6]. In short, we prepared two samples (in 50 mM Tris, 100 mM NaCl, 7.5% D_2_O, 30 μM DSS, pH 7.4) containing 200 μM ^15^N-enriched ubiquitin and either no or a 5 x molar excess of the unlabeled Ube2S (residues 1–156) Cys 118 variant and recorded phase-sensitive gradient-enhanced ^1^H-^15^N HSQC spectra [51]. A weighted combined chemical shift difference, Δδ(^1^H^15^N), was calculated according to
Δδ(H1N15)=(δ(H1)−δ(H1)0)2+0.04(δ(N15)−δ(N15)0)2
where δ(^1^H) and δ(^15^N) denote the chemical shifts in the presence of Ube2S, and δ(^1^H)_0_ and δ(^15^N)_0_ denote the chemical shifts in the absence of Ube2S, respectively. The weighted combined chemical shift differences are plotted. Ubiquitin interacts with all tested Ube2S variants in a similar way (Figure A). In vitro activity assays monitoring diubiquitin formation by Ube2S (residues 1–156). We compared reactions in the absence (-) and presence (+) of ATP. All three variants are active in diubiquitin formation, but display reduced activity compared to the wildtype; the activity of the C118M variant is closest to the wildtype level. The reduced activities of the Cys 118 variants are not due to a loss of donor binding (Figure B).(PDF)

S2 File Analysis of lattice contacts of ubiquitin in the crystal structure of the Ube2S-ubiquitin conjugate. Overview of all molecules that ubiquitin (green) contacts in the context of the crystal lattice. The molecules are shown in cartoon representation along with the symmetry operations that should be applied to ubiquitin in order to obtain the respective interface (specified in fractional space relative to the structure position given in the PDB file), the interface area (calculated as the difference in the total accessible surface areas of the isolated and interfacing structures and divided by 2), and the solvation free energy gain upon formation of the interface, Δ^i^G (calculated as the difference in the total solvation energies of the isolated and the interfacing structures), according to the PDBePISA server (www.ebi.ac.uk/pdbe/pisa). The Ube2S molecule to which ubiquitin (green) is linked covalently is shown in yellow, the Ube2S molecule, with which ubiquitin forms the hydrophobic, closed trans interface is shown in orange (Figure A). Detailed view of the crystallographic interface between ubiquitin (green) and a neighboring Ube2S molecule (grey). Contacting side chains are displayed in ball-and-stick representation (Figure B).
(PDF)

S3 File Comparison of the closed Ube2S-ubiquitin interface with other closed E2-donor complexes. Superposition of the Ube2S-ubiquitin configuration seen in our crystal structure in trans with the closed UbcH5A-ubiquitin conjugate bound to the RING domain dimer of RNF4 (PDB ID: 4AP4) [10]. Note that the second RNF4-RING subunit is bound to another E2-conjugate in the crystal structure that is not displayed here (Figure A). Superposition of the Ube2S-ubiquitin configuration seen in our crystal structure (in trans) with a non-covalent, closed Cdc34-ubiquitin complex bound to an inhibitor (PDB ID: 4MDK; the inhibitor is not displayed) [12] (Figure B). Interaction “footprints” on the surface of ubiquitin in the crystal structures of the three E2-donor complexes displayed in Figure A and Figure B, as defined by residues that become ≥ 10% buried at the interface. The contacting surface areas on ubiquitin are very similar. Note that we truncated the C-terminal tail of ubiquitin in our Ube2S-ubiquitin complex (PDB ID: 5BNB) for this representation due to the closed interface being formed in trans (Figure C).
(PDF)

S1 Table Detailed comparison of interfacial residues in the crystal structure with the docked model as well as NMR- and mutagenesis data. The NMR data and Haddock input parameters are taken from [6]. According to Haddock nomenclature, NMR-based ambiguous interaction restraints that were used for the docking calculations were subdivided into “active” and “passive” residues [46]. Active residues were specified based on a chemical shift perturbation of the corresponding NMR resonance, Δδ(^1^H^15^N), of > 0.1 ppm in the presence of a ~ 6 x molar excess of ubiquitin and Ube2S, respectively, and > 35% surface accessibility of the residue [6]. Passive residues were defined as surface neighbors of active residues within a distance of 6.5 Å with > 50% surface accessibility, as previously described [6]. The analysis of the interfaces in the docked model and the crystal structure is based on the PDBePISA server (www.ebi.ac.uk/pdbe/pisa). Note that residues 101 and 102 of Ube2S that make contacts in the docked model, but not in the crystallographic interface are close to the active site and, therefore, contact residues near the ubiquitin tail that is oriented towards the E2 active site in the docked model, but mis-oriented in the crystal structure, where we interpret an interface between two molecules in trans. The listed results of the diubiquitin formation assays are taken from the present and our previous study [6].
(TIF)
