# Supplementary figures and images for: Crystal Structure of a Ube2S-Ubiquitin Conjugate
Source: PLoS One. 2016 Feb 1;11(2):e0147550. doi: 10.1371/journal.pone.0147550 (PMC4734694; doi:10.1371/journal.pone.0147550)

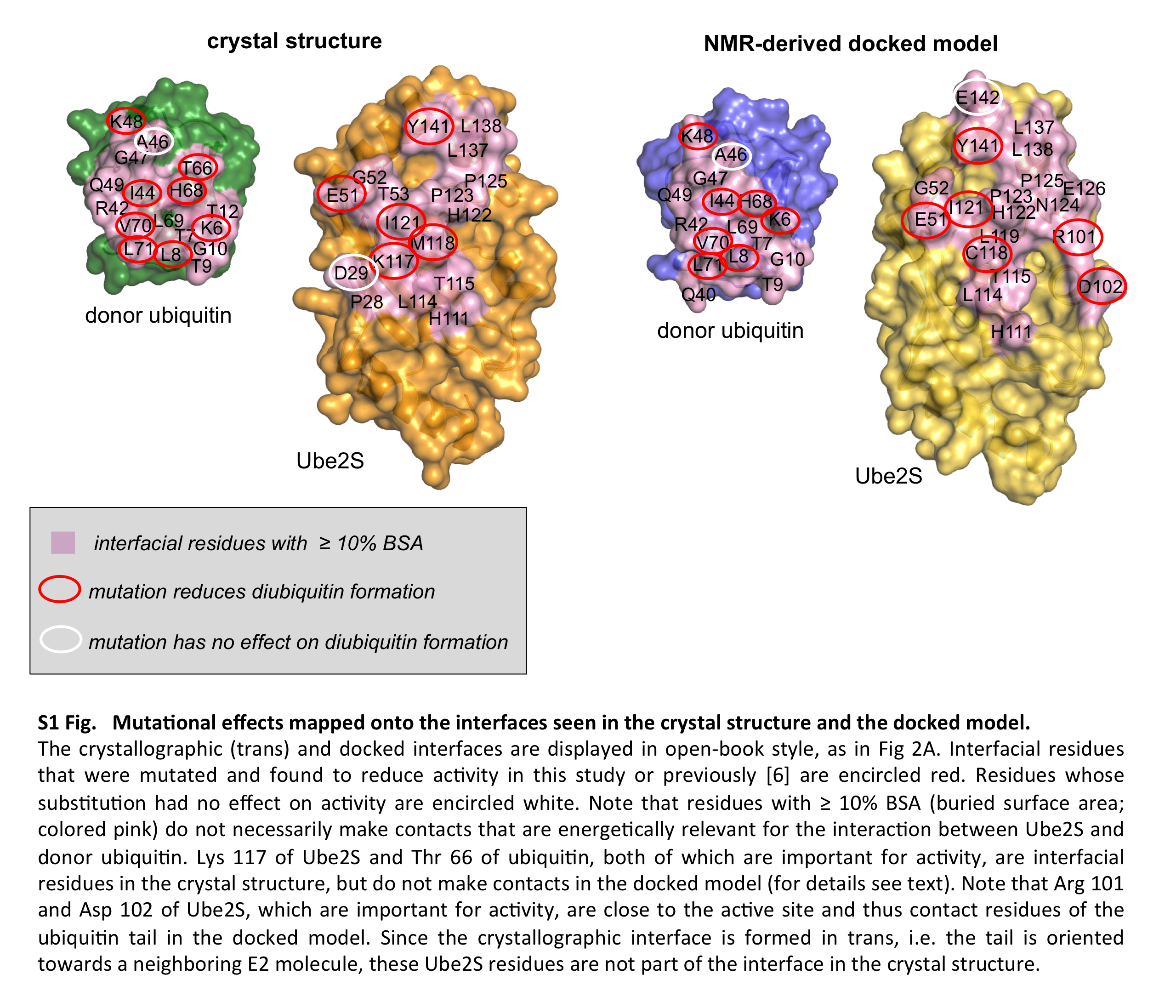

Supplement: S1 Fig — The crystallographic (trans) and docked interfaces are displayed in open-book style, as in Fig 2A. Interfacial residues that were mutated and found to reduce activity in this study or previously [6] are encircled red. Residues whose substitution had no effect on activity are encircled white. Note that residues with ≥ 10% BSA (buried surface area; colored pink) do not necessarily make contacts that are energetically relevant for the interaction between Ube2S and donor ubiquitin. Lys 117 of Ube2S and Thr 66 of ubiquitin, both of which are important for activity, are interfacial residues in the crystal structure, but do not make contacts in the docked model (for details see text). Note that Arg 101 and Asp 102 of Ube2S, which are important for activity, are close to the active site and thus contact residues of the ubiquitin tail in the docked model. Since the crystallographic interface is formed in trans, i.e. the tail is oriented towards a neighboring E2 molecule, these Ube2S residues are not part of the interface in the crystal structure. (TIF) [file pone.0147550.s001.tif]

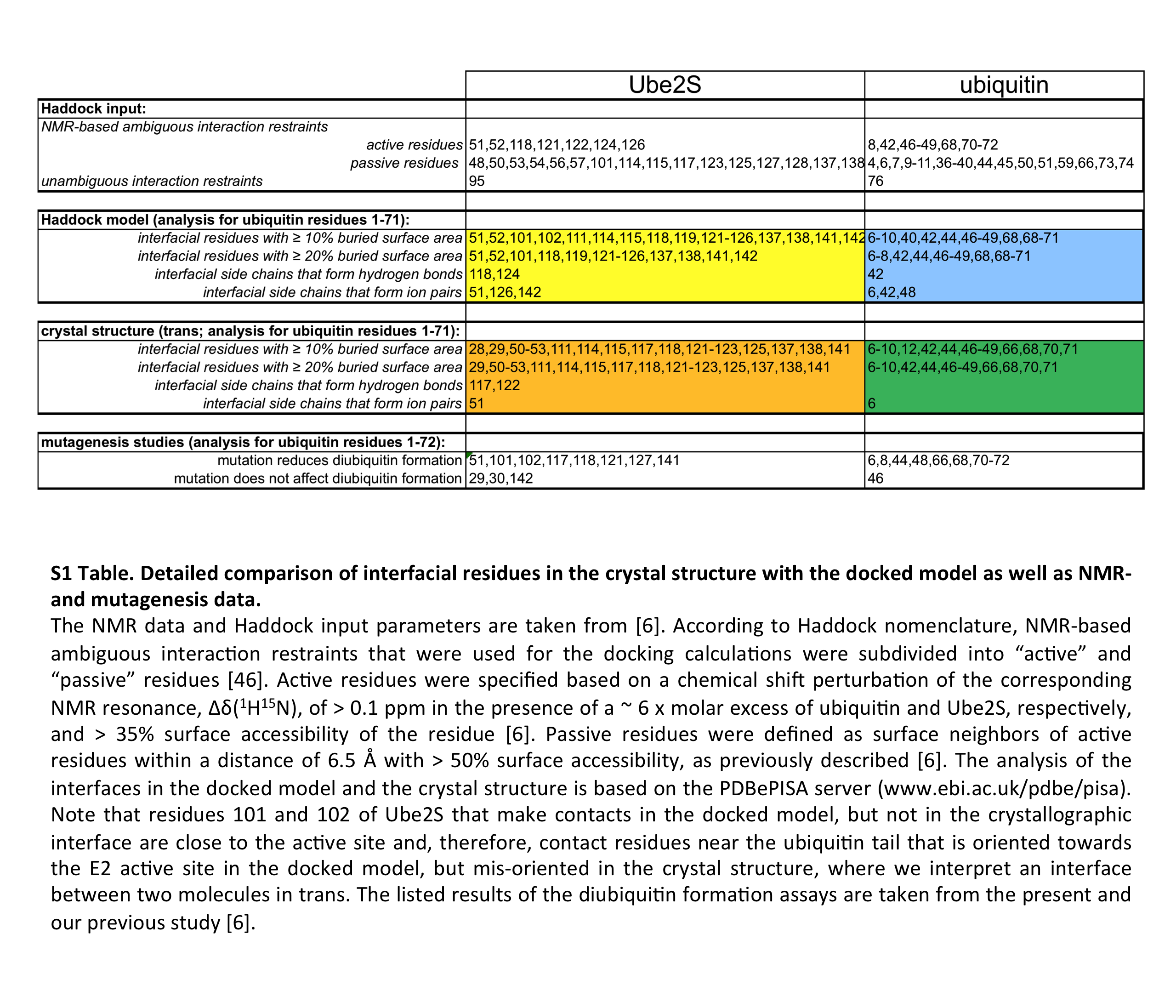

Supplement: S1 Table — The NMR data and Haddock input parameters are taken from [6]. According to Haddock nomenclature, NMR-based ambiguous interaction restraints that were used for the docking calculations were subdivided into “active” and “passive” residues [46]. Active residues were specified based on a chemical shift perturbation of the corresponding NMR resonance, Δδ(1H15N), of > 0.1 ppm in the presence of a ~ 6 x molar excess of ubiquitin and Ube2S, respectively, and > 35% surface accessibility of the residue [6]. Passive residues were defined as surface neighbors of active residues within a distance of 6.5 Å with > 50% surface accessibility, as previously described [6]. The analysis of the interfaces in the docked model and the crystal structure is based on the PDBePISA server (www.ebi.ac.uk/pdbe/pisa). Note that residues 101 and 102 of Ube2S that make contacts in the docked model, but not in the crystallographic interface are close to the active site and, therefore, contact residues near the ubiquitin tail that is oriented towards the E2 active site in the docked model, but mis-oriented in the crystal structure, where we interpret an interface between two molecules in trans. The listed results of the diubiquitin formation assays are taken from the present and our previous study [6]. (TIF) [file pone.0147550.s005.tif]
