# Supplementary material for: Crystal Structure of a Ube2S-Ubiquitin Conjugate
Source: PLoS One. 2016 Feb 1;11(2):e0147550. doi: 10.1371/journal.pone.0147550 (PMC4734694; doi:10.1371/journal.pone.0147550)
Supplement: S1 File — NMR data were recorded at 25°C on a Bruker 800 MHz DRX spectrometer, equipped with a 1H/15N/13C cryoprobe and were processed with NMRPipe [50]. The binding experiments were performed as described previously [6]. In short, we prepared two samples (in 50 mM Tris, 100 mM NaCl, 7.5% D2O, 30 μM DSS, pH 7.4) containing 200 μM 15N-enriched ubiquitin and either no or a 5 x molar excess of the unlabeled Ube2S (residues 1–156) Cys 118 variant and recorded phase-sensitive gradient-enhanced 1H-15N HSQC spectra [51]. A weighted combined chemical shift difference, Δδ(1H15N), was calculated according to Δδ(H1N15)=(δ(H1)−δ(H1)0)2+0.04(δ(N15)−δ(N15)0)2 where δ(1H) and δ(15N) denote the chemical shifts in the presence of Ube2S, and δ(1H)0 and δ(15N)0 denote the chemical shifts in the absence of Ube2S, respectively. The weighted combined chemical shift differences are plotted. Ubiquitin interacts with all tested Ube2S variants in a similar way (Figure A). In vitro activity assays monitoring diubiquitin formation by Ube2S (residues 1–156). We compared reactions in the absence (-) and presence (+) of ATP. All three variants are active in diubiquitin formation, but display reduced activity compared to the wildtype; the activity of the C118M variant is closest to the wildtype level. The reduced activities of the Cys 118 variants are not due to a loss of donor binding (Figure B). (PDF) [file pone.0147550.s002.pdf]

Figure A

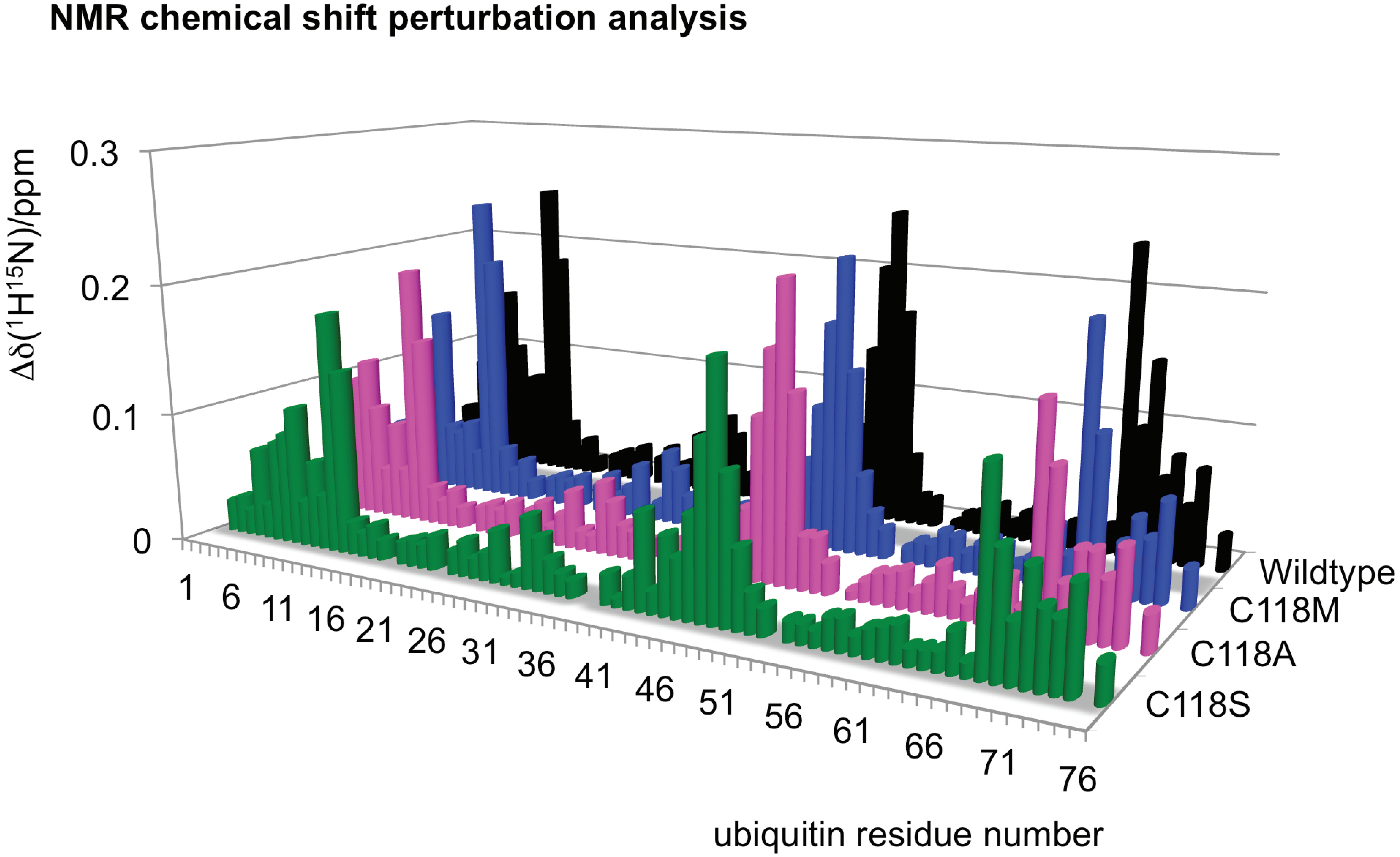

Figure B

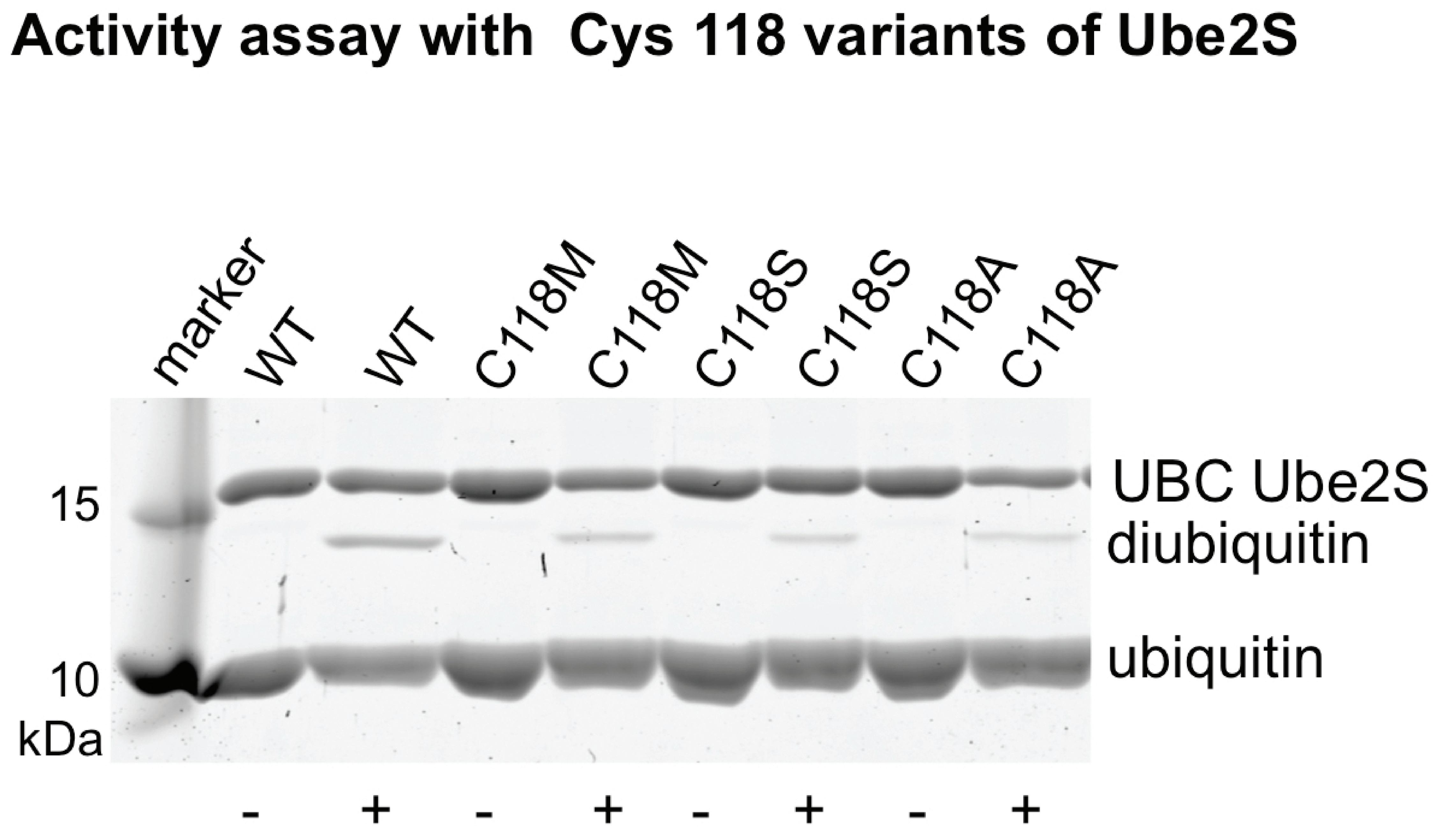

S1 File. Effects of Cys 118 substitutions in Ube2S on ubiquitin binding and activity.

NMR data were recorded at 25 °C on a Bruker 800 MHz DRX spectrometer, equipped with a $^{1}H/^{15}N/^{13}C$ cryoprobe and were processed with NMRPipe [50]. The binding experiments were performed as described previously [6]. In short, we prepared two samples (in 50 mM Tris, 100 mM NaCl, 7.5% D_2O, 30 µM DSS, pH 7.4) containing 200 µM ^{15}N-enriched ubiquitin and either no or a 5 x molar excess of the unlabeled Ube2S (residues 1-156) Cys 118 variant and recorded phase-sensitive gradient-enhanced ^{1}H-^{15}N HSQC spectra [51]. A weighted combined chemical shift difference, $\Delta\delta(^{1}H^{15}N)$, was calculated according to $\Delta\delta(^{1}H^{15}N)=[(\delta(^{1}H)-\delta(^{1}H)_0)^2+0.04(\delta(^{15}N)-\delta(^{15}N)_0)^2]^{0.5}$, where $\delta(^{1}H)$ and $\delta(^{15}N)$ denote the chemical shifts in the presence of Ube2S, and $\delta(^{1}H)_0$ and $\delta(^{15}N)_0$ denote the chemical shifts in the absence of Ube2S, respectively. The weighted combined chemical shift differences are plotted. Ubiquitin interacts with all tested Ube2S variants in a similar way (Figure A).

In vitro activity assays monitoring diubiquitin formation by Ube2S (residues 1-156). We compared reactions in the absence (-) and presence (+) of ATP. All three variants are active in diubiquitin formation, but display reduced activity compared to the wildtype; the activity of the C118M variant is closest to the wildtype level. Note that the reduced activities of the Cys 118 variants are not due to a loss of donor binding (Figure B).
