# Supplementary material for: Crystal Structure of a Ube2S-Ubiquitin Conjugate
Source: PLoS One. 2016 Feb 1;11(2):e0147550. doi: 10.1371/journal.pone.0147550 (PMC4734694; doi:10.1371/journal.pone.0147550)
Supplement: S2 File — Overview of all molecules that ubiquitin (green) contacts in the context of the crystal lattice. The molecules are shown in cartoon representation along with the symmetry operations that should be applied to ubiquitin in order to obtain the respective interface (specified in fractional space relative to the structure position given in the PDB file), the interface area (calculated as the difference in the total accessible surface areas of the isolated and interfacing structures and divided by 2), and the solvation free energy gain upon formation of the interface, ΔiG (calculated as the difference in the total solvation energies of the isolated and the interfacing structures), according to the PDBePISA server (www.ebi.ac.uk/pdbe/pisa). The Ube2S molecule to which ubiquitin (green) is linked covalently is shown in yellow, the Ube2S molecule, with which ubiquitin forms the hydrophobic, closed trans interface is shown in orange (Figure A). Detailed view of the crystallographic interface between ubiquitin (green) and a neighboring Ube2S molecule (grey). Contacting side chains are displayed in ball-and-stick representation (Figure B). (PDF) [file pone.0147550.s003.pdf]

Figure A

Lattice contacts in the crystal of the Ube2S-ubiquitin conjugate (PDB ID: 5BNB)

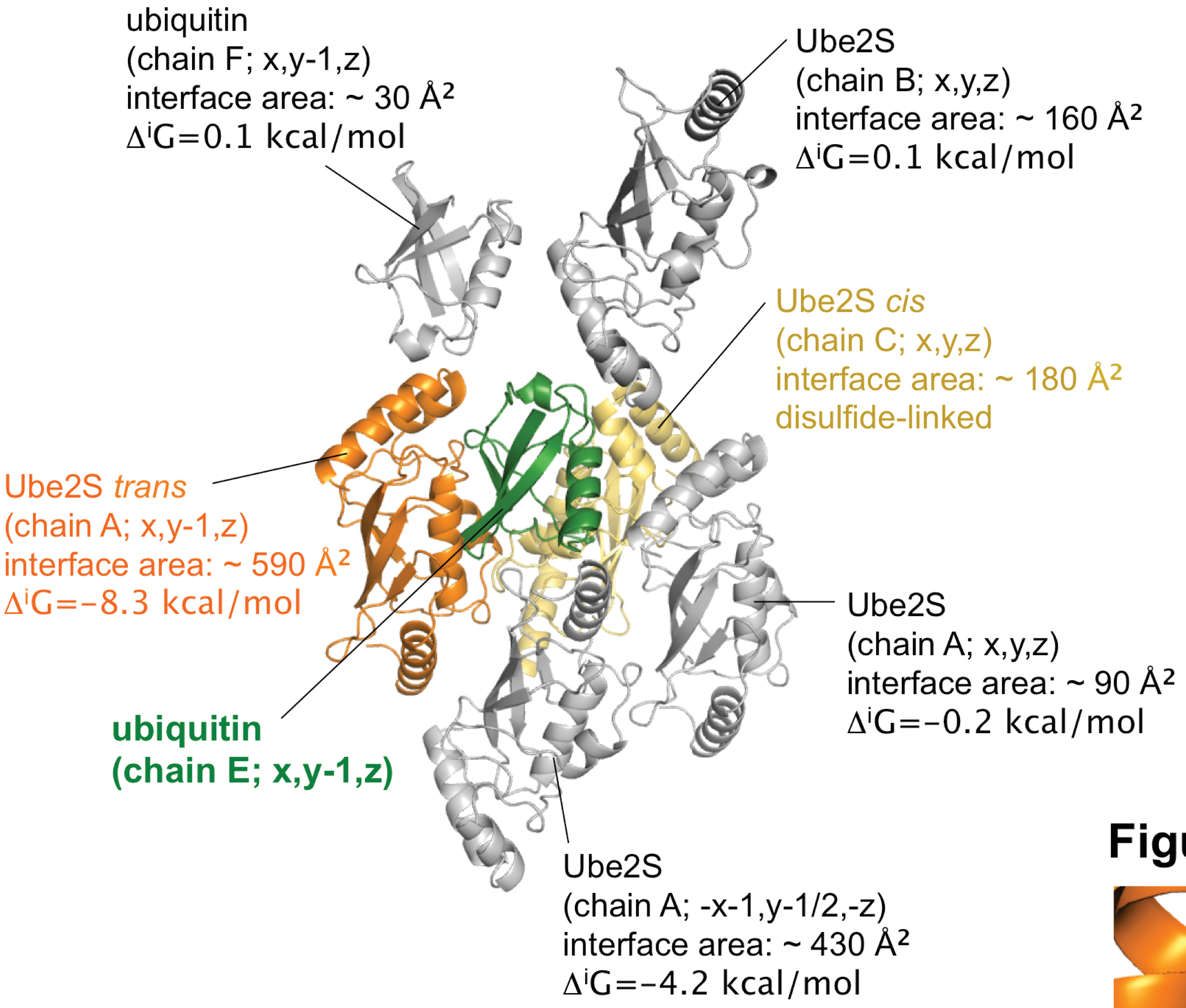

Figure B

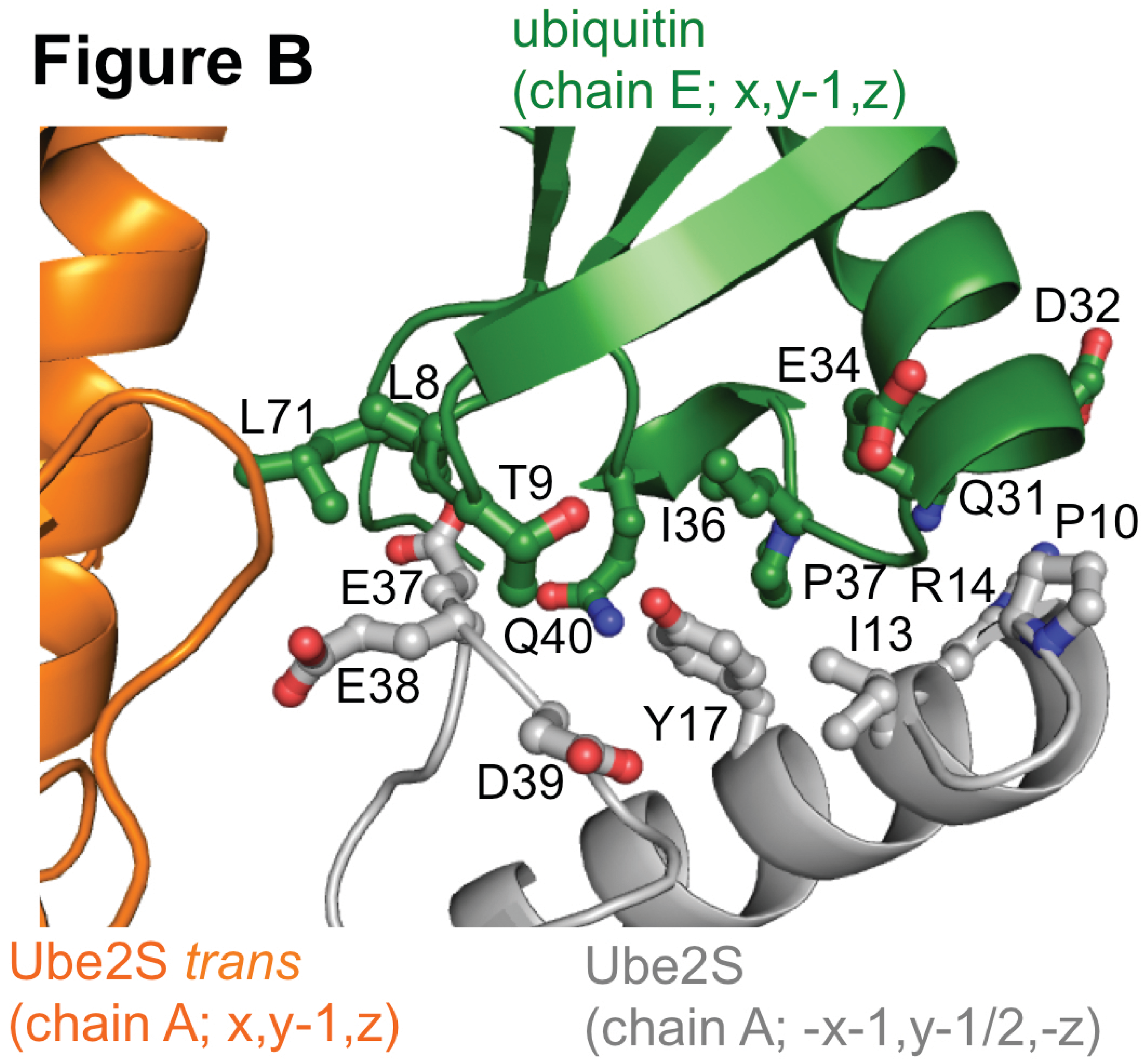

S2 File. Analysis of lattice contacts of ubiquitin in the crystal structure of the Ube2S-ubiquitin conjugate.

Overview of all molecules that ubiquitin (green) contacts in the context of the crystal lattice. The molecules are shown in cartoon representation along with the symmetry operations that should be applied to ubiquitin in order to obtain the respective interface (specified in fractional space relative to the structure position given in the PDB file), the interface area (calculated as the difference in the total accessible surface areas of the isolated and interfacing structures and divided by 2), and the solvation free energy gain upon formation of the interface, $\Delta^{i}G$ (calculated as the difference in the total solvation energies of the isolated and the interfacing structures), according to the PDBePISA server (www.ebi.ac.uk/pdbe/pisa). The Ube2S molecule to which ubiquitin (green) is linked covalently is shown in yellow, the Ube2S molecule, with which ubiquitin forms the hydrophobic, closed trans interface is shown in orange (Figure A). Detailed view of the crystallographic interface between ubiquitin (green) and a neighboring Ube2S molecule (grey). Contacting side chains are displayed in ball-and-stick representation (Figure B).
