# Supplementary material for: Crystal Structure of a Ube2S-Ubiquitin Conjugate
Source: PLoS One. 2016 Feb 1;11(2):e0147550. doi: 10.1371/journal.pone.0147550 (PMC4734694; doi:10.1371/journal.pone.0147550)
Supplement: S3 File — Superposition of the Ube2S-ubiquitin configuration seen in our crystal structure in trans with the closed UbcH5A-ubiquitin conjugate bound to the RING domain dimer of RNF4 (PDB ID: 4AP4) [10]. Note that the second RNF4-RING subunit is bound to another E2-conjugate in the crystal structure that is not displayed here (Figure A). Superposition of the Ube2S-ubiquitin configuration seen in our crystal structure (in trans) with a non-covalent, closed Cdc34-ubiquitin complex bound to an inhibitor (PDB ID: 4MDK; the inhibitor is not displayed) [12] (Figure B). Interaction “footprints” on the surface of ubiquitin in the crystal structures of the three E2-donor complexes displayed in Figure A and Figure B, as defined by residues that become ≥ 10% buried at the interface. The contacting surface areas on ubiquitin are very similar. Note that we truncated the C-terminal tail of ubiquitin in our Ube2S-ubiquitin complex (PDB ID: 5BNB) for this representation due to the closed interface being formed in trans (Figure C). (PDF) [file pone.0147550.s004.pdf]

Figure A

Superposition of PDB ID 5BNB with 4AP4

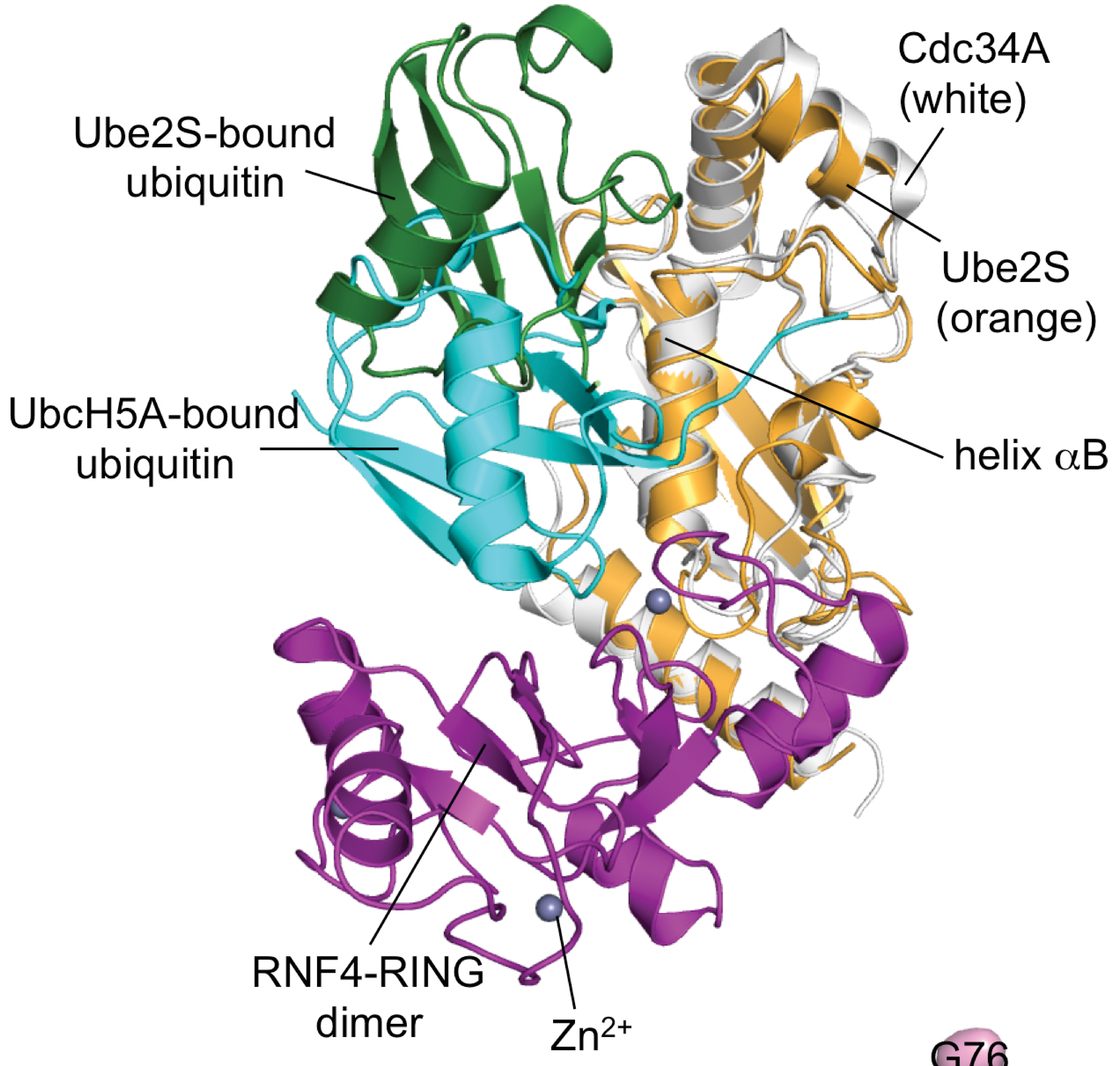

Figure B

Superposition of PDB ID 5BNB with 4MDK

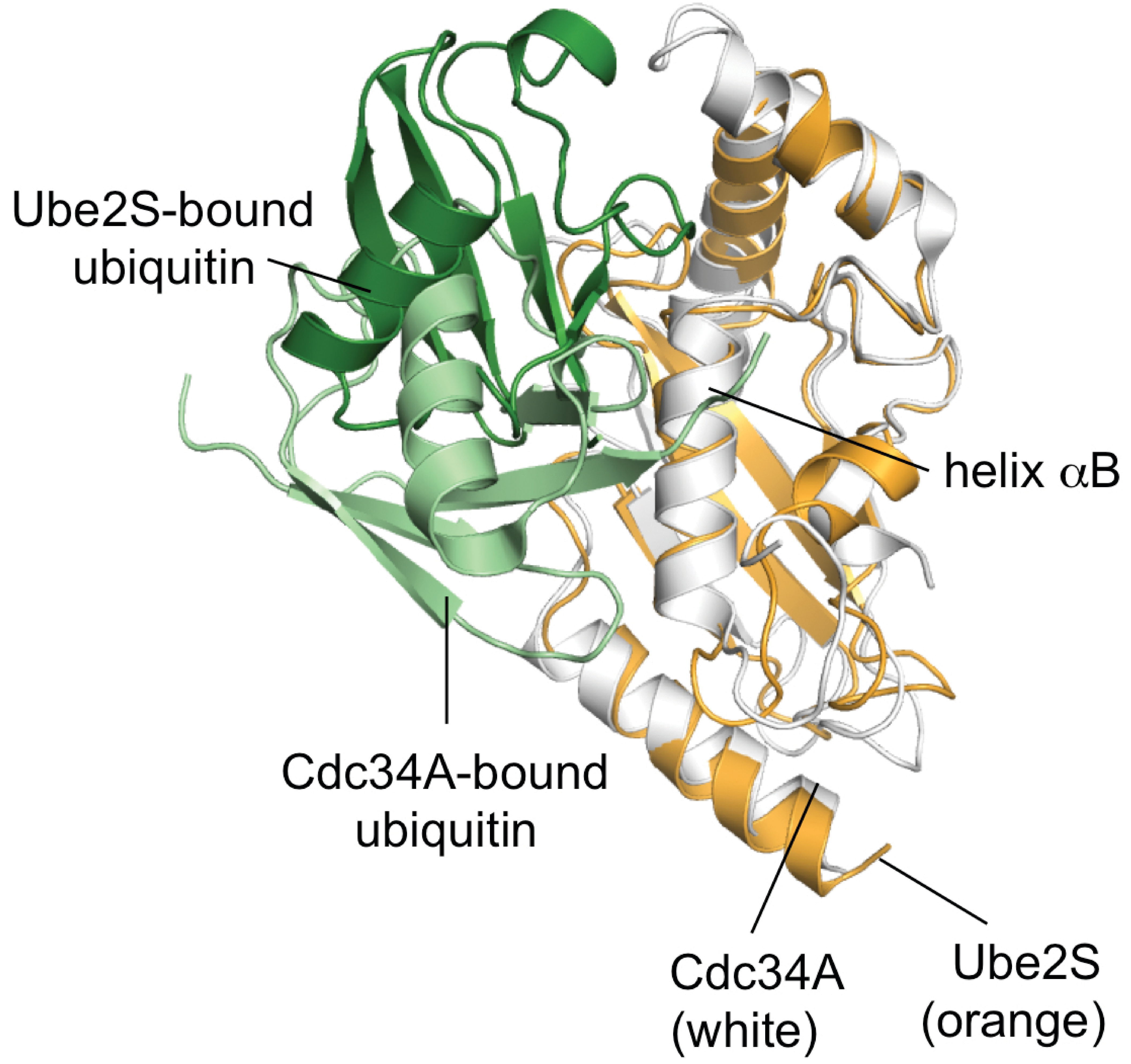

Figure C

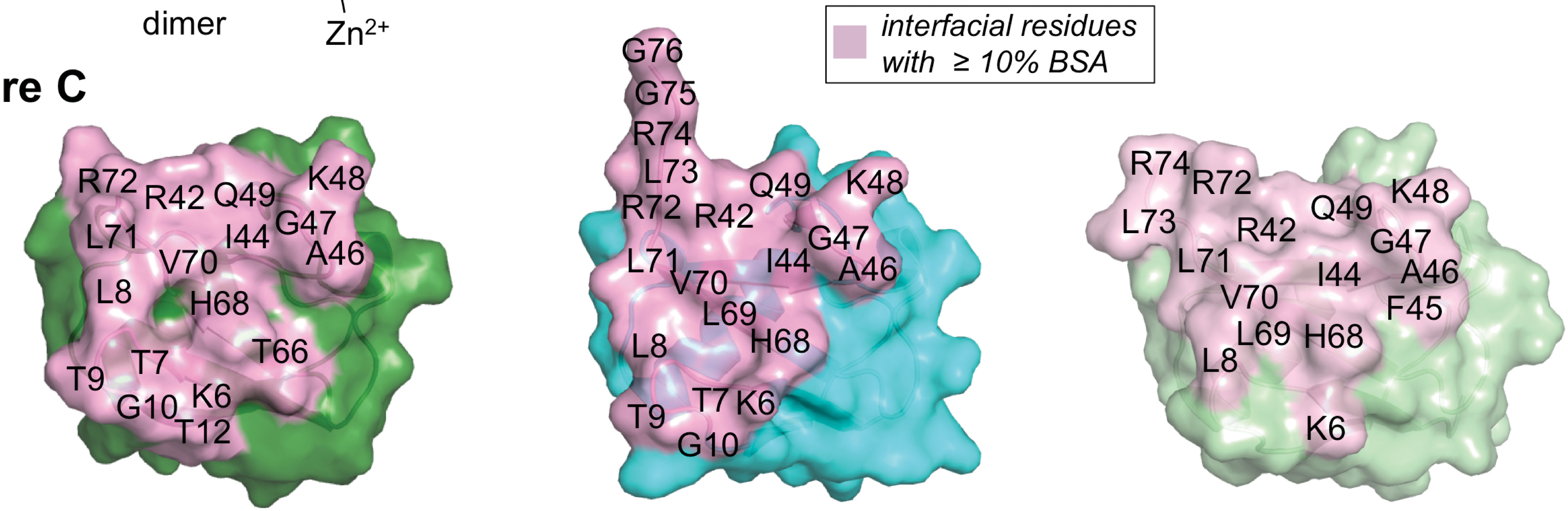

Ube2S-ubiquitin complex
(PDB ID:5BNB, trans interface)

UbcH5A-ubiquitin-RNF4 RING complex
(PDB ID: 4AP4)

Cdc34-ubiquitin complex
(PDB ID: 4MDK)

S3 File. Comparison of the closed Ube2S-ubiquitin interface with other closed E2-donor complexes.

Superposition of the Ube2S-ubiquitin configuration seen in our crystal structure in trans with the closed UbcH5A-ubiquitin conjugate bound to the RING domain dimer of RNF4 (PDB ID: 4AP4) [10]. Note that the second RNF4-RING subunit is bound to another E2-conjugate in the crystal structure that is not displayed here (Figure A). Superposition of the Ube2S-ubiquitin configuration seen in our crystal structure (in trans) with a non-covalent, closed Cdc34-ubiquitin complex bound to an inhibitor (PDB ID: 4MDK; the inhibitor is not displayed) [12] (Figure B). Interaction "footprints" on the surface of ubiquitin in the crystal structures of the three E2-donor complexes displayed in Figure A and Figure B, as defined by residues that become ≥ 10% buried at the interface. The contacting surface areas on ubiquitin are very similar. Note that we truncated the C-terminal tail of ubiquitin in our Ube2S-ubiquitin complex (PDB ID: 5BNB) for this representation due to the closed interface being formed in trans (Figure C).
